# Extended-wavelength diffuse reflectance spectroscopy with a machine-learning method for *in vivo* tissue classification

**DOI:** 10.1371/journal.pone.0223682

**Published:** 2019-10-10

**Authors:** Ulf Dahlstrand, Rafi Sheikh, Cu Dybelius Ansson, Khashayar Memarzadeh, Nina Reistad, Malin Malmsjö

**Affiliations:** 1 Lund University, Skåne University Hospital, Department of Clinical Sciences Lund, Ophthalmology, Lund, Sweden; 2 Department of Atomic Physics, Lund University, Lund, Sweden; Politecnico di Milano, ITALY

## Abstract

**Objectives:**

An extended-wavelength diffuse reflectance spectroscopy (EWDRS) technique was evaluated for its ability to differentiate between and classify different skin and tissue types in an *in vivo* pig model.

**Materials and methods:**

EWDRS recordings (450–1550 nm) were made on skin with different degrees of pigmentation as well as on the pig snout and tongue. The recordings were used to train a support vector machine to identify and classify the different skin and tissue types.

**Results:**

The resulting EWDRS curves for each skin and tissue type had a unique profile. The support vector machine was able to classify each skin and tissue type with an overall accuracy of 98.2%. The sensitivity and specificity were between 96.4 and 100.0% for all skin and tissue types.

**Conclusion:**

EWDRS can be used *in vivo* to differentiate between different skin and tissue types with good accuracy. Further development of the technique may potentially lead to a novel diagnostic tool for e.g. non-invasive tumor margin delineation.

## Introduction

Histopathological examination is currently the gold standard in diagnosing skin cancer. The tumor can either be histologically examined after or during the course of the operation, or surgery can be conducted in two steps separated by two days, during which the tumor is examined. Complete circumferential peripheral and deep margin assessment is the preferred method for the removal of complicated skin tumors, and involves histological sectioning that allows for the complete examination of the surgical margin of the specimen [1]. This is an invasive technique that is time-consuming. Furthermore, if the tumor is not radically excised, with margins free of tumor tissue, there may be a need for reoperation. There is thus a need for faster and non-invasive techniques for tumor margin delineation.

A number of non-invasive techniques are being used to provide information on skin lesions prior to histopathological analysis, each with its own advantages and disadvantages. *Dermoscopy* is carried out with a hand-held device that provides magnification of the skin, enabling visualization of microstructures from the epidermis down to the papillary dermis. The information obtained in this way can be used to classify cutaneous cancer [2, 3] and significantly better diagnostic accuracy has been demonstrated with dermoscopy compared to conventional clinical examination [4]. However, it is a subjective modality and requires a trained, experienced user to obtain reliable results.

*Reflectance confocal microscopy* is a laser-based method that allows examination down to the depth of the papillary dermis with a resolution that is close to that of histological analyses. In a meta-analysis, the pooled sensitivity for the diagnosis of several skin cancers was found to be 93.2%, but the specificity was only 82.8%. The accuracy of the method also seems to be dependent on the experience of the observer [5].

*High-frequency ultrasound* uses ultrasound from 20 to 100 MHz to obtain real-time images of the tumor. As most skin tumors are hypoechogenic, its primary use is not in providing a reliable diagnosis, but rather in giving a pre-surgical indication of tumor thickness and its potential infiltrative properties [6]. Other emerging modalities, such as *optical coherence tomography*, are also being evaluated [7].

There is currently no rapid, non-invasive method of diagnosing skin tumors that does not depend on the experience of the user. *Diffuse reflectance spectroscopy* (DRS) is an optical method that uses a light source connected to a fiberoptic probe to illuminate the tissue. The diffusely reflected light reaching the probe after interaction with the tissue is then collected and analyzed. The intensity of the reflected light varies with the concentration of chromophores in the tissue. A spectrometer is used to display the intensity of the reflected light as a function of its wavelength [8] and commonly used spectrometers capture light in the visible to near-infrared range (VIS-NIR; 400 to 1000 nm) or the near-infrared to short-wave infrared range (NIR-SWIR; 1000 to 1700 nm).

We have recently developed a DRS system that combines two spectrometers (VIS-NIR and NIR-SWIR) to visualize an *extended-wavelength spectrum*, between 450 and 1550 nm [9–11]. Melanin and hemoglobin (both oxygenated and deoxygenated) are the most significant chromophores, and absorb most of the light in the VIS-NIR. The strongest absorbers in the NIR-SWIR region are water and lipids, but collagen also shows prominent absorption peaks [12]. The combination of these two spectrometers thus covers many of the important components of human and animal skin. Measurements over this extended range of wavelengths provide more information than conventional DRS, and studies have shown that the ability to correctly quantify water, lipids and blood content can be significantly improved [12]. It has previously been shown that this extended-wavelength DRS technique (EWDRS) can be used to accurately differentiate between healthy and metastatic human liver tissue [13, 14], evaluate liver steatosis grade [15] and to monitor perfusion in porcine eyelid flaps [9]. Our hope is to be able to further develop this EWDRS technique into a tool for non-invasive detection and classification of skin cancers, which would not dependent on the experience of the user.

The present study was carried out to evaluate the ability of EWDRS to differentiate between five different skin and tissue types in an *in vivo* pig model. The aim was to determine whether it is possible to use statistical methods and machine learning to correctly classify each tissue type using EWDRS. Specimens were also analyzed histologically in order to compare the histological appearance with the EWDRS findings.

## Materials and methods

### Animals and anesthesia

Eight domestic pigs with an average bodyweight of 72 kg (range 53–88 kg) were used in this study. They were fasted overnight, but had free access to water. An intramuscular injection of dexmedetomidine (Dexdomitor 0.5 mg/ml, Orion Pharma AB Animal Health, Sollentuna, Sweden, 0.03 mg/kg) mixed with tiletamine/zolazepam (Zoletil 100 Vet. 100 mg/ml, Virbac, Nice, France, 6 mg/kg) was used for premedication. Anesthesia was then induced by intravenous sodium thiopental (Pentocur, Abcur AB, Helsingborg, Sweden, 0.5 g mixed with 20 ml 0.9% sodium chloride solution) and fentanyl (Fentanyl B. Braun, Melsungen, Germany, 2 μg/kg), and maintained by continuous infusion of fentanyl in Ringer’s acetate (3.5 μg/kg·h) in combination with sodium thiopental (~2.5 mg/kg). The animals were orally intubated with cuffed endotracheal tubes. Mechanical ventilation was established in the volume-controlled mode with 35% oxygen (Siemens-Elema AB, Solna, Sweden). The ventilation settings were identical for all animals: respiratory rate 15 breaths/min and minute ventilation 12 l/min. A positive end-expiratory pressure of 5 cm H_2_O was applied. A Foley catheter was inserted into the urinary bladder through a suprapubic cystostomy. The flanks were shaved in order to allow skin measurements to be made. Following anesthesia and the surgical procedures, the pig was allowed to stabilize for 1 hour before the experiments were started. At the end of the experiments, the pigs were euthanized while still under general anesthesia using barbiturate pentobarbital (Euthasol vet 400 mg/ml, Virbac, Carros, France).

### Ethics

The experimental protocol for this study was approved by the Ethics Committee for Animal Research at Lund University, Sweden (protocol number M154-13). All animals received humane care in compliance with the European Convention on Animal Care. The animals were also used for other experiments, which were considered not to have an impact on the present study.

### Experimental procedure

The tissues examined were:

- non-pigmented skin on the flank,
- semi-pigmented skin on the flank,
- heavily pigmented skin on the flank,
- the snout, and
- the tongue.

All skin and tissue types were studied in each pig, except for one pig, where only the first three measurements were made. All pigs had large patches with semi-pigmented skin and within these areas there were smaller patches of heavily pigmented skin. EWDRS measurements were made at several different locations for each tissue type on each pig, and three to five consecutive recordings were made at each location.

Samples from each tissue type were excised and sent for histological analysis.

### Diffuse reflectance spectroscopy

Diffuse reflectance spectral signatures were collected using a portable spectroscopic system comprising a contact fiber optic probe, a tungsten halogen light source (HL-2000-HP, Ocean Optics, Dunedin, FL, USA) providing a broadband spectrum from around 360 nm to 2000 nm, and two miniature spectrometers. The two spectrometers resolve light in the VIS-NIR wavelength range from 350 nm to 1100 nm (QE65000-VIS-NIR, Ocean Optics) with 1044 x 64 pixels, and in the NIR region from 900 nm to 1700 nm (NIRQuest512, Ocean Optics) using 512 pixels. The VIS-NIR spectrometer has a 50 μm slit providing approximately 3 nm optical resolution, and the NIR spectrometer a 25 μm slit, also providing about 3 nm optical resolution. The probe is a 10-mm-diameter trifurcated fiber bundle consisting of a 10-fiber, 200-μm-diameter signal-collection ring around a single illuminating 400-μm-diameter fiber with a source–detector separation of 2.5 mm. The distal end of the probe is fixed in a custom-made black cylindrical probe holder with a diameter of 25 mm, which is held against the tissue to stabilize the probe–tissue contact and to block out other light sources such as ambient light. Reference spectra were acquired before and after each set of tissue measurements using a reflectance standard (Spectralon SRS-99-010, Labsphere, North Sutton, NH, USA). Data acquisition from the spectrometers was controlled by Ocean-View software (Ocean Optics) on a standard laptop computer. Measurements were made directly on the surface of each kind of tissue. All raw tissue spectra were background calibrated and intensity normalized. The relevant overlapping wavelength region of both spectrometers (900 to 1100 nm) was used to compute a merging factor that ideally should be equal to one, and this was used to combine the two spectra into a single continuous spectrum. Due to light source limitations and to be able to ensure a good signal-to-noise ratio, the final wavelength range was set to 450 to 1550 nm. Interpolation was used to give the spectrum a 1 nm resolution. [10]

### Histology

Representative specimens of each tissue type were surgically excised and immediately fixed in 4% paraformaldehyde. After fixation for some weeks, the tissues were dehydrated and embedded in paraffin. Thereafter, the tissues were placed in 70% ethanol for 36 hours before dehydration and paraffin embedding. The tissues were embedded using a Tissue Tek VIP E150 tissue processor (Sakura, Tokyo, Japan) overnight at room temperature through the following steps: 40 minutes in 70% ethanol, 2x1 hour in 96% ethanol, 2x1 hour in 99.9% ethanol and 2x1 hour in xylene before 3x1 hour in 100% paraffin at 60°C. The samples were then placed in 100% paraffin at 60°C for 2 hours. The samples were then embedded in 100% paraffin blocks (Tissue Tek Mega-Cassette system, Sakura).

Tissue blocks were sectioned in 5 μm sections with a rotating microtome (HM360, Microm International GmbH, Walldorf, Germany), and the sections placed on microscope slides (Menzel-Gläser, Braunschweig, Germany). The sections were then allowed to dry at room temperature overnight, and were then heated at 60°C for 1 hour prior to deparaffinization by soaking for 5 min in xylene, 5 min in 99.5% ethanol, and 5 min in distilled water. After deparaffinization histological staining was performed with Fontana-Masson stain (Melanin stain ab150669, Abcam, Cambridge, MA, USA) to visualize melanin. The tissue sections were stained according to the protocol provided with the Fontana-Masson kit, first for 30 min in ammoniacal silver solution pre-warmed to 58°C until the sections turned yellow/brown. Sections were then washed several times in distilled water before being stained for 30 seconds with 0.2% gold chloride solution, followed by washing in distilled water several times, and then incubation in sodium thiosulfate for 2 minutes. The sections were then washed in running tap water and then washed twice in distilled water. Finally, the sections were stained with nuclear fast red for 5 min, washed in running tap water and then washed twice in distilled water. Before mounting with Pertex mounting medium (Histolab, Gothenburg, Sweden) the sections were dehydrated, first quickly 3 times in 99.9% ethanol and then in xylene.

The snout and tongue samples were stained with hematoxylin-eosin stain according to the standard procedure after the same tissue treatment as for the skin samples described above.

### Calculations and statistics

#### Diffuse reflectance spectroscopy

Three to five EWDRS recordings were made at each location for every tissue type, and the average was used for further calculations (Table 1). If the recordings differed significantly upon graphical visualization, they were discarded, as this indicated that the probe was not being held steadily. In total, 28 recordings of 639 were discarded.

Principal component analysis (PCA) was used to reduce the dimensions of the data in order to be able to construct predictive classification models. It is characterized by an orthogonal transformation of the original data set onto a reduced subspace spanned by the principal components. By choosing the number of principal components to be five, 99.4% of the total variance of all data was still represented.

**Table 1:** The number of averaged DRS measurements recorded for each tissue type.

Tissue type	Total number of measurements	Number of pigs
Non-pigmented skin	135	8
Semi-pigmented skin	135	8
Heavily pigmented skin	126	8
Snout	112	7
Tongue	103	7

#### Machine learning and classification

Different types of machine-learning models were assessed, such as linear discriminant analysis, decision trees, k-nearest neighbor classification and support vector machines (SVM). SVM using a quadratic kernel was found to give the best accuracy and was selected for further use. The method was validated using stratified five-fold cross-validation, meaning that the data were randomly divided into five groups of equal sizes, and four of these were used to train the model, and the last one to test it. This it then repeated for all folds, and the average test error is used to evaluate the model. All five principal components were used as predictors, and the true tissue type was used as the response parameter. Sensitivity and specificity for each tissue type in comparison to the others combined was calculated using Matlab R2016b (The MathWorks Inc., South Natick, MA, USA) from a confusion matrix. Furthermore, the overall estimated accuracy was calculated. The 95% confidence interval was determined using Wilson score interval. Due to imbalanced data across the different classes, micro- and macro-averaged F-scores were calculated, as well as the baseline performance of a majority classifier. The overall accuracy was also calculated when using only the data from 450 to 900 nm, and the results were compared to that from the full range of wavelengths (450 to 1550 nm). Spectrum processing and data classification were performed using Matlab R2016b.

## Results

### DRS measurements and histology

**Fig 1:**
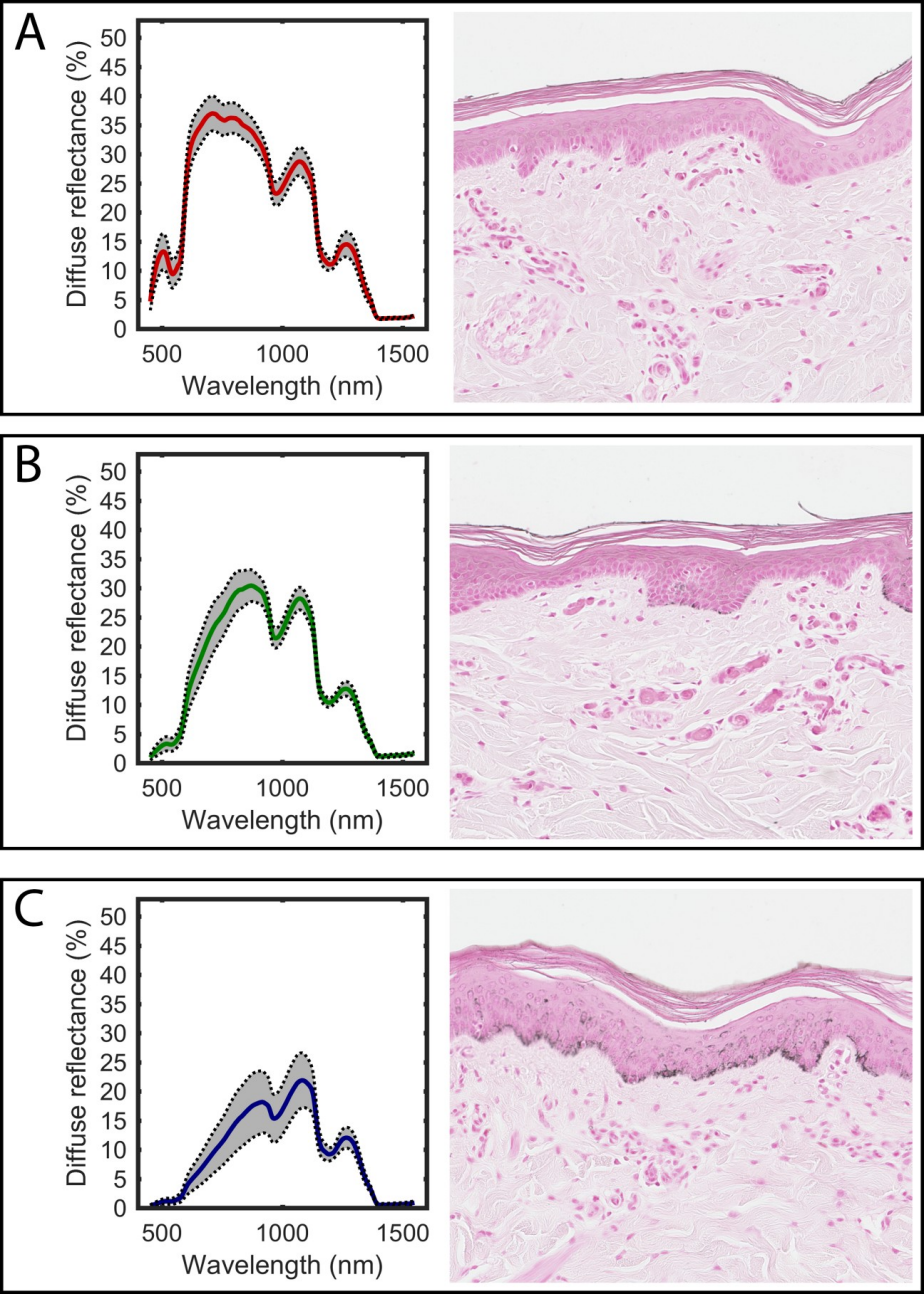
The average diffuse reflectance curves for the different skin types. A) non-pigmented skin, B) semi-pigmented skin, and C) heavily pigmented skin. The standard deviation is indicated by the gray shading. Representative corresponding histological images are shown on the right (original magnification x 20).

**Fig 2:**
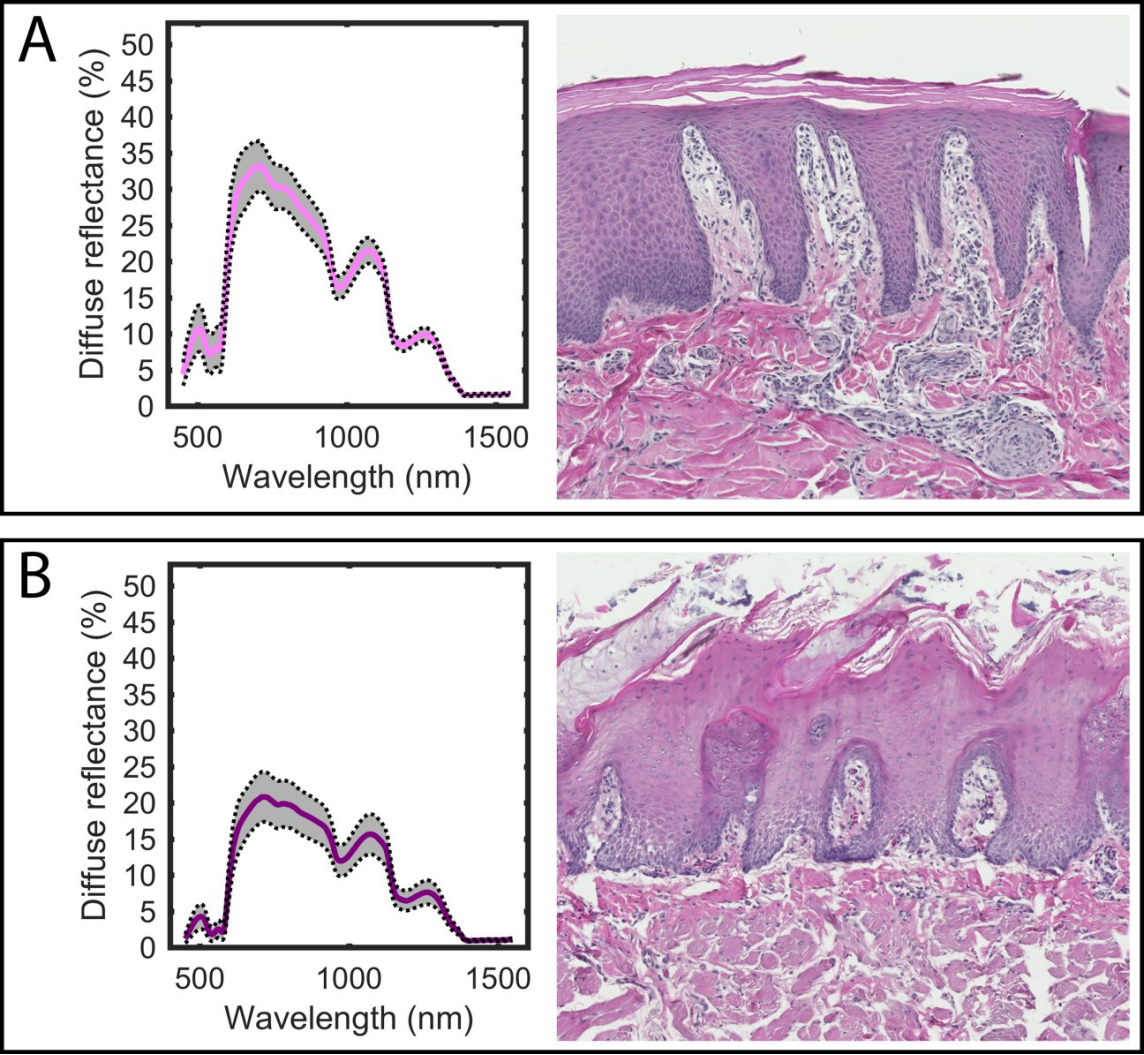
**The average diffuse reflectance curves for A) snout and B) tongue.** The standard deviation is indicated by the gray shading. Representative corresponding histological images are shown on the right (original magnification x 10).

The five different skin and tissue types produced unique spectral reflectance responses when illuminated with the same light source. The average DRS curves are shown in Figs 1 and 2. In the skin measurements (Fig 1), it is clear that the reflected signal in the VIS-NIR region decreases with increasing pigmentation, being most pronounced at the shorter wavelengths. This may be due to the increasing melanin content in the basal part of the epidermis, as can be seen in the histological images. The signals from the snout and tongue (Fig 2) differ in that the tongue shows generally lower amplitudes in the VIS-NIR region. This is probably due to the fact that the tongue is a muscular organ that contains high amounts of blood. Both oxygenated and deoxygenated hemoglobin are strong absorbers in this wavelength region.

### Classification

**Fig 3:**
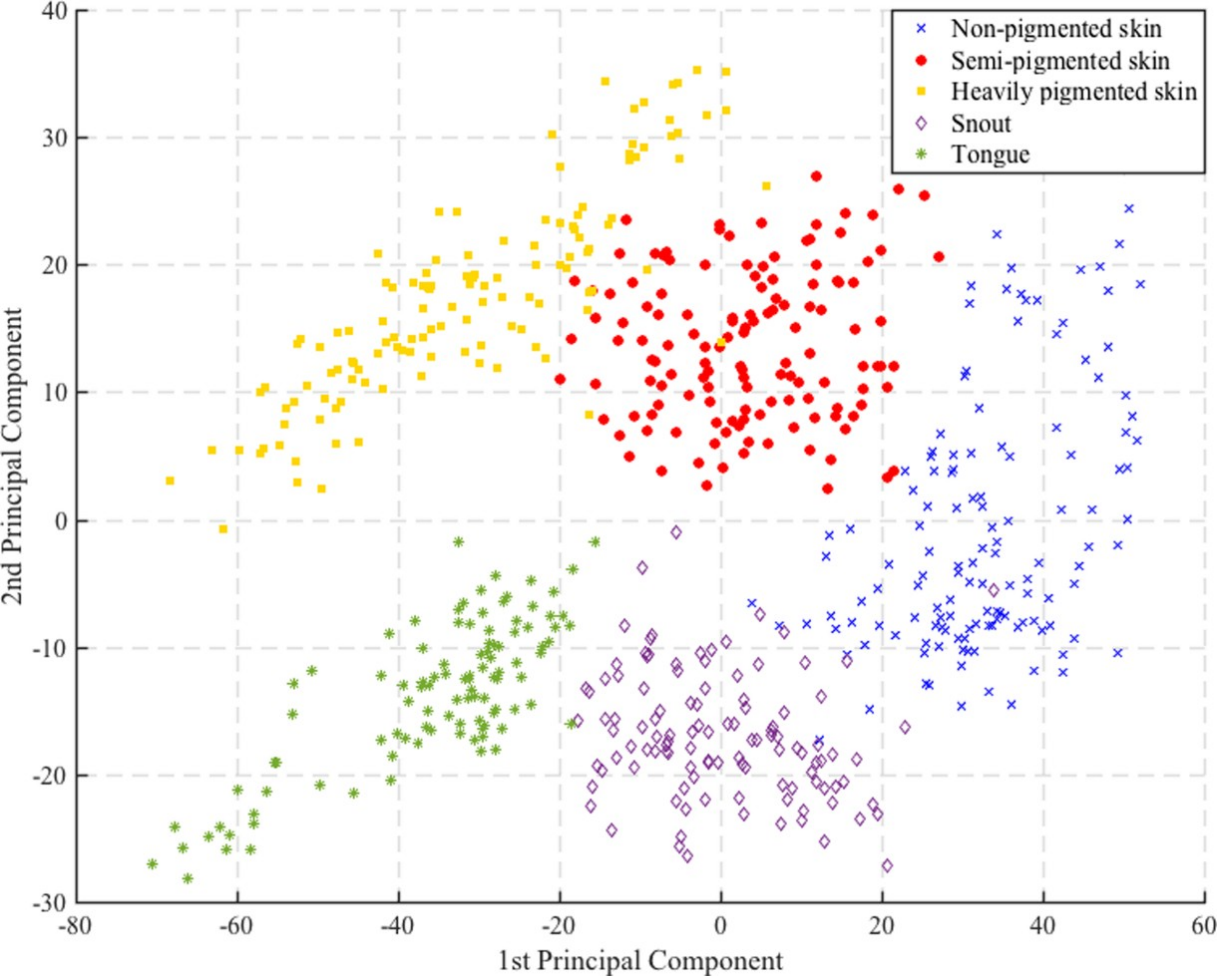
The EWDRS measurements displayed using the first and second principal component after PCA. The five different tissue types appear as clusters with little overlap.

The EWDRS signals were transformed using PCA, and a graphical representation of the first and second principal components indicated that the different tissue type could be distinguished (Fig 3).

Using the quadratic SVM with the five-fold cross-validation method it was possible to classify the DRS measurements into the five different tissue types with an overall estimated accuracy of 98.2%. The sensitivity and specificity of one tissue type in comparison to the others combined, are given in Table 2. The micro- and macro averaged F scores were 98.1% and 98.2% respectively. Running a majority classifier on the same data for comparison gave an overall accuracy of only 22.1%. When using only the information from 450 to 900 nm, a similar overall accuracy of 98.7% was achieved.

**Table 2:** The sensitivity and specificity, expressed as %, for each tissue type in comparison to the others combined (95% CI).

Tissue type	Sensitivity	Specificity
Non-pigmented skin	98.6 (94.8–99.6)	99.8 (98.8–100.0)
Semi-pigmented skin	98.4 (94.8–99.6)	99.4 (98.2–99.8)
Heavily pigmented skin	98.3 (94.4–99.6)	99.6 (98.5–99.9)
Snout	96.4 (91-2-98.6)	99.6 (98.6–99.9)
Tongue	99.0 (94.7–99.8)	99.4 (98.3–99.8)

## Discussion

This paper describes the first step in an attempt to develop a non-invasive method to differentiate between and classify different skin and tissue types. Five kinds of animal tissue that were easily accessible, and expected to have different optical properties, were used to test the potential of EWDRS. The pig is considered to be a suitable animal for skin studies, as the epidermis, dermis, and subcutaneous fat resemble those of humans [16, 17]. Thus, we examined porcine skin with different levels of pigmentation as well as the snout and tongue.

DRS can be used for tissue diagnosis as it provides information by analyzing the reflected light after interaction with the tissue. Every type of tissue has a unique combination of chromophores, and the DRS signal thus provides an optical “fingerprint” of the tissue. In order to obtain as much information as possible, we have constructed an extended-wavelength DRS device, in which the signal is collected at wavelengths from 450 to 1550 nm, using a single probe. Using this device, we found that each skin and tissue type studied exhibited a unique spectral signature. This is in line with previous studies showing that DRS could be used to determine properties such as the hemoglobin and melanin concentration in skin [18], the oxygen saturation in skin flaps [19], and to classify breast cancer biopsies [20], by collecting spectra in the UV-VIS or the VIS-NIR region. Other studies have focused on the SWIR region, where water, lipids, and collagen are strong absorbers [21].

We then used PCA to preprocess the data and, with the SVM learning model, it was possible to classify the recordings with an overall accuracy of over 98%, with good sensitivity and specificity for all tissue types. SVM is a method of automatic classification that has been widely applied in the field of medicine, for example, to classify cancers based on tumor markers in the blood [22], to interpret electroencephalography signals [23], to determine subgroups of schizophrenia [24], and to aid in decision-making in patients with symptoms of acute coronary syndrome [25].

When examining the selected tissues used in this study, it was found that similar accuracy could be obtained using only the VIS-NIR-spectrometer. This could probably be explained by melanin being the dominant chromophore in this wavelength range and that three skin types with different degrees of pigmentation was examined. Indeed, in the present study there was no advantage gained by extending the wavelength range from 450–900 nm to 450–1550 nm and this brings into question the significance of the work. However, in future tissue and tumor examinations, there could be other important chromophores that might require using the full wavelength range for detection. It is our hope that the EWDRS technique could be used to differentiate between, for example, healthy skin and pathological skin lesions. This would however, require in depth studies on tumor tissue and surrounding healthy tissue as well as clinical trials. It would be of great value to be able to correctly diagnose and pre-surgically define tumor margins of malignant melanomas and non-pigmented skin lesions such as basal cell carcinomas, squamous cell carcinomas and actinic keratosis.

## Conclusion

In conclusion, EWDRS can be used *in vivo* to differentiate between different skin and tissue types with excellent specificity and sensitivity. The SVM learning model allowed for skin and tissue type classification with an overall accuracy of over 98%. EWDRS examination can be performed with a hand-held probe and takes only a few seconds. Using suitable software, the signal could be analyzed in real time to provide a non-observer-dependent diagnosis.

Further development of the technique may lead to a novel diagnostic tool for e.g. non-invasive tumor classification and margin delineation.

## Supporting information

S1 All spectra graphs All measured spectra for the different tissue types, visualized as graphs. (DOCX)

S1 All spectra data All measured preprocessed spectra for the different tissue types, as a .mat file. (MAT)
