# Supplementary figures and images for: Extended-wavelength diffuse reflectance spectroscopy with a machine-learning method for in vivo tissue classification
Source: PLoS One. 2019 Oct 10;14(10):e0223682. doi: 10.1371/journal.pone.0223682 (PMC6786558; doi:10.1371/journal.pone.0223682)

S1 All extended-wavelength diffuse reflectance spectroscopy spectra
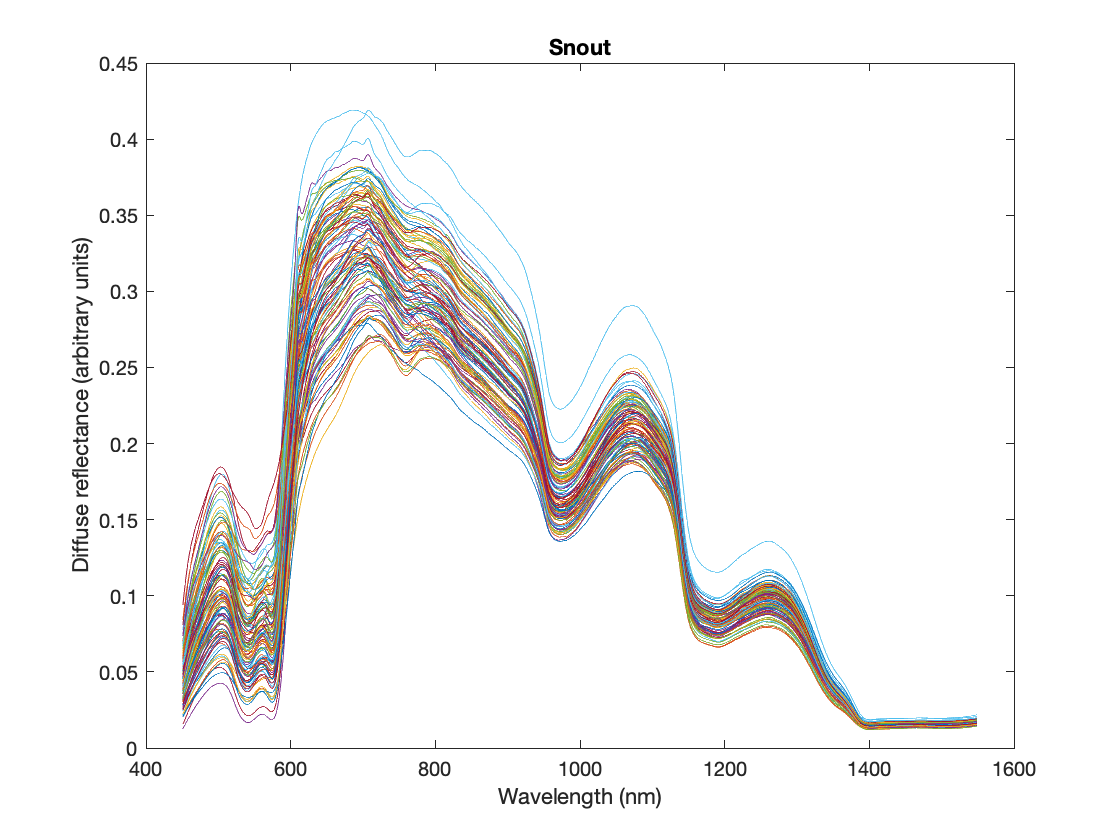

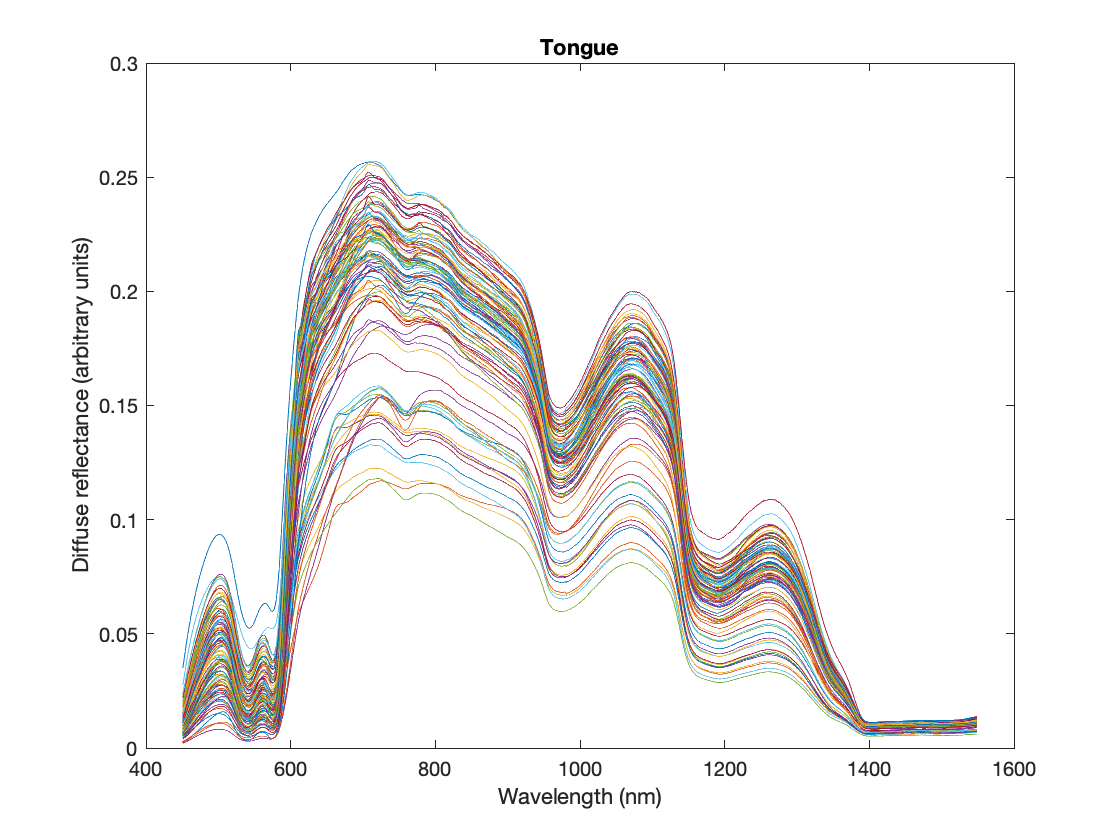

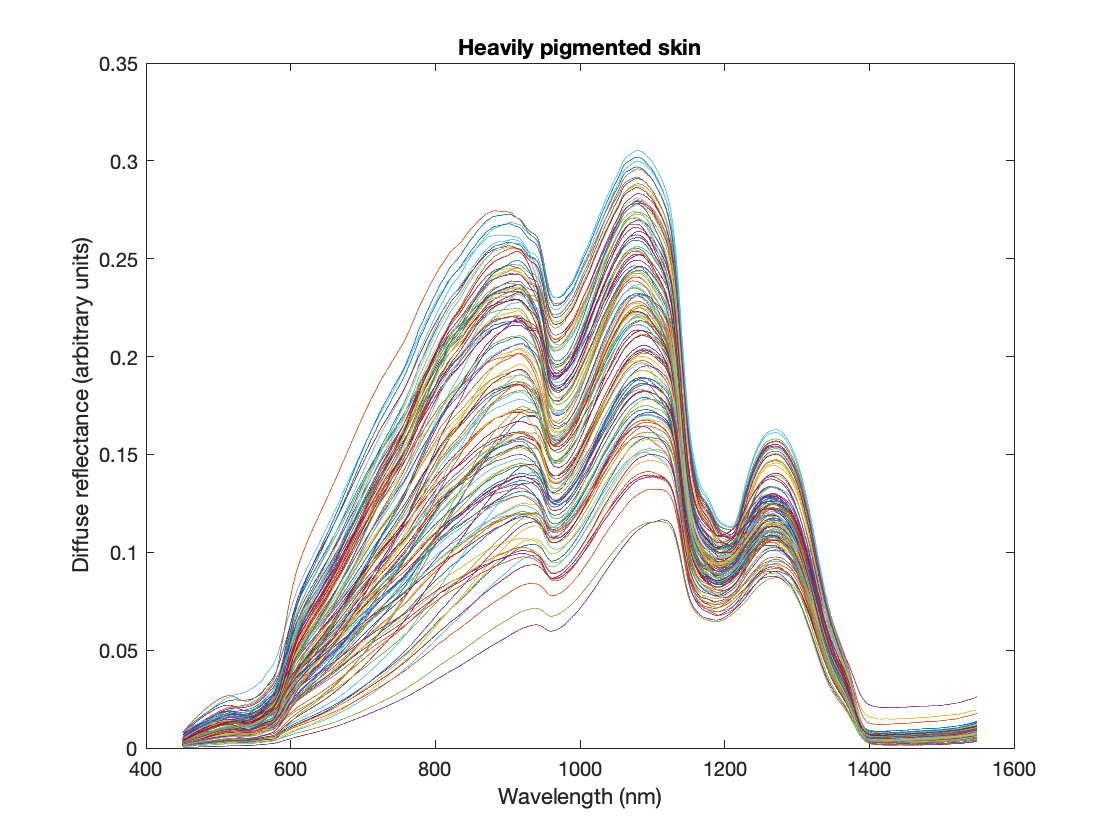


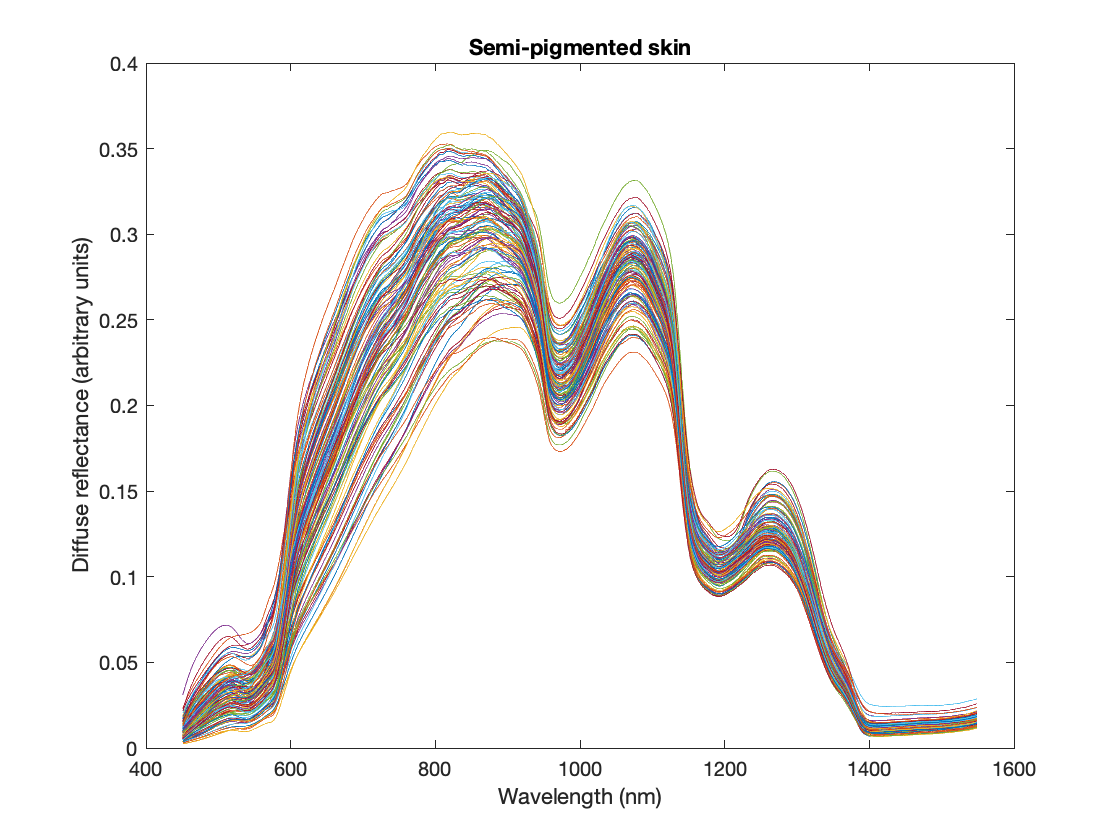


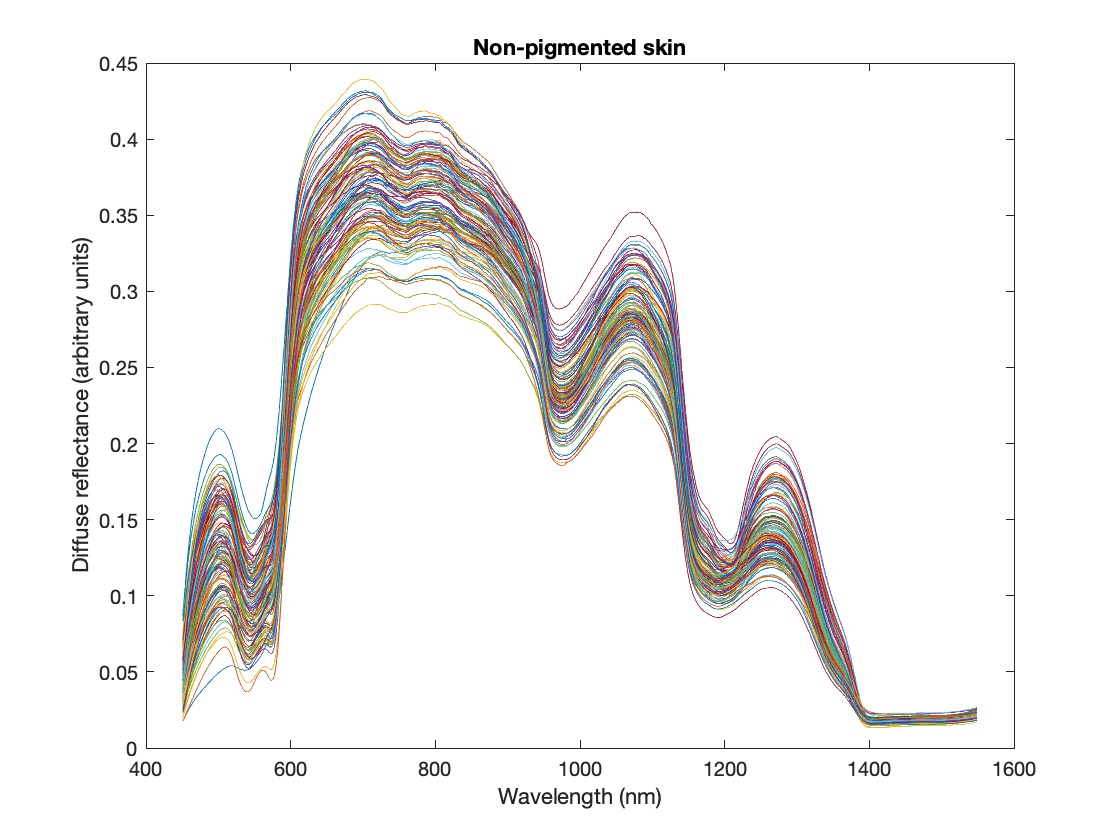

Supplement: S1 All spectra graphs — (DOCX) [file pone.0223682.s001.docx]
